# Resting-state subcortical functional connectivity in HIV-infected patients on long-term cART

**DOI:** 10.1007/s11682-016-9632-4

**Published:** 2016-10-15

**Authors:** Marloes A. M. Janssen, Max Hinne, Ronald J. Janssen, Marcel A. van Gerven, Stefan C. Steens, Bozena Góraj, Peter P. Koopmans, Roy P. C. Kessels

**Affiliations:** 1 Department of Medical Psychology, Radboud University Medical Center, P.O. box 9101, 6500 HB Nijmegen, the Netherlands; 2 Donders Institute for Brain, Cognition and Behaviour, Radboud University, Nijmegen, the Netherlands; 3 Institute for Computing and Information Sciences, Radboud University, Nijmegen, the Netherlands; 4 Department of Radiology and Nuclear Medicine, Radboud University Medical Center, Nijmegen, the Netherlands; 5 Department of Diagnostic Imaging, Medical Center for Postgraduate Education, Warsaw, Poland; 6 Department of Internal Medicine, Radboud University Medical Center, Nijmegen, the Netherlands

**Keywords:** HIV, Magnetic resonance imaging, Resting-state, Subcortical functional connectivity, Bayesian method, Partial correlations

## Abstract

Despite long-term successful treatment with cART, impairments in cognitive functioning are still being reported in HIV-infected patients. Since changes in cognitive function may be preceded by subtle changes in brain function, neuroimaging techniques, such as resting-state functional magnetic resonance imaging (rs-fMRI) have become useful tools in assessing HIV-associated abnormalities in the brain. The purpose of the current study was to examine the extent to which HIV infection in virologically suppressed patients is associated with disruptions in subcortical regions of the brain in comparison to a matched HIV-negative control group. The sample consisted of 72 patients and 39 controls included between January 2012 and January 2014. Resting state functional connectivity was determined between fourteen regions-of-interest (ROI): the left and right nucleus accumbens, amygdala, caudate nucleus, hippocampus, putamen, pallidum and thalamus. A Bayesian method was used to estimate resting-state functional connectivity, quantified in terms of partial correlations. Both groups showed the strongest partial correlations between the left and right caudate nucleus and the left and right thalamus. However, no differences between the HIV patients and controls were found between the posterior expected network densities (control network density = 0.26, SD = 0.05, patient network density = 0.26, SD = 0.04*, p = 0.58)*. The results of the current study show that HIV does not affect subcortical connectivity in virologically controlled patients who are otherwise healthy.

## Background

The potential effects of human immunodeficiency virus (HIV) infection on the brain remain a major concern in the combination anti-retroviral therapy (cART) era, as impairments in cognitive functioning are still being reported in HIV-infected patients, despite long-term successful treatment (Heaton et al. 2011; Cysique and Brew 2011; Tozzi et al. 2007; Simioni et al. 2010; Janssen et al. 2015). Cognitive functioning is commonly assessed by neuropsychological testing. However, changes in cognitive function may be preceded by subtle changes in brain function. Neuroimaging techniques, such as resting-state functional magnetic resonance imaging (rs-fMRI) have become useful tools in assessing HIV-associated abnormalities in the brain. Rs-fMRI is a method that is used to evaluate the intrinsic temporal covariation of the blood-oxygen-level dependent (BOLD) signal acquired when a participant is not performing an explicit task. Using this technique, Thomas et al. (2013) investigated five functional brain networks including the default mode, dorsal attention, salience, executive control and sensorimotor networks in HIV-infected patients. Lower intra-network correlations were found in the default mode and control and salience networks compared to HIV-negative controls. However, a study by Wang et al. (2011), investigating rs-fMRI networks in HIV-infected patients within their first year of HIV infection, only reported impaired functional connectivity in the lateral occipital cortex network. No differences were reported in other resting-state networks, including the default mode network and executive control. Another study used rs-fMRI to investigate the frontostriatal network and found that HIV status and cognitive impairment were associated with a lower connectivity between the dorsal caudate and the dorsolateral prefrontal cortex (Ipser et al. 2015). Also, Ortega et al. (2015) showed that HIV-infected patients had lower functional connectivity between the striatum and the default mode network and ventral attention network compared to HIV-negative controls, with HIV-infected patients on cART showing higher connectivity between these networks than patients not on cART. Thames et al. (2016) found increased activation in basal ganglia structures (e.g. the putamen and caudate) in HIV-infected patients during a phonemic fluency task. Furthermore, alterations have been demonstrated in various networks, such as the default mode, salience network and frontal and motor networks in older HIV-infected patients (Jahanshad et al. 2012; Thomas et al. 2013; Guha et al. 2016). To summarize, all studies mentioned above investigated frontal areas, for example, the executive control network, fronto-striatal network, and the frontal subcortical network. Some, but not all studies also examined the default mode network and sensorimotor areas (e.g., Thomas et al. 2013; Wang et al. 2011 and Ortega et al. 2015). Abnormalities in the default mode network are reported in several studies (Thomas et al. 2013; Ortega et al. 2015), but not in patients within their first year of infection (Wang et al. 2011).

The most consistent finding in previous functional connectivity studies on HIV is that differences are found between HIV-infected patients and healthy controls in executive control networks (Thomas et al. 2013; Ipser et al. 2015; Ortega et al. 2015; Thames et al. 2016). However, previous resting-state fMRI studies focused exclusively on cortical and cortico-subcortical functional connectivity. So far, functional connectivity between subcortical structures, especially the basal ganglia, hippocampus, amygdala and thalamus, has not been studied. This is relevant, as structural abnormalities in these brain regions were found in HIV-infected patients in some but not all studies (Janssen et al. 2015; Wade et al. 2015; Samuelsson et al. 2006; Towgood et al. 2013). Potentially, functional connectivity measures may be more sensitive to HIV-related abnormalities, which can be hypothesized to be small especially in successfully treated HIV-infected patients, and as such complement structural neuroimaging findings (Rosazza and Minati 2011).

Also, few rs-fMRI studies only included HIV-infected patients on stable cART. Ortega et al. (2015) differentiated their results into three groups: a HIV infected group on cART, a patient group not on cART and a control group. Thomas et al. (2013) controlled for the effect of treatment (treatment did not have an effect on functional connectivity in their study). The inclusion in Thames et al. (2016) was limited to patients on cART, but they investigated subcortical connectivity only in a small study sample (12 HIV- and 16 HIV+ patients) and only in relation to a very specific cognitive task (i.e., phonemic fluency). Since most current HIV patients in the Western world are being treated with cART, studies focusing on virologically suppressed HIV patients increase the external validity of the results.

Hence, the purpose of the current study was to examine the extent to which HIV infection in virologically suppressed patients on cART is associated with disruptions in specific subcortical regions of the brain in comparison to a matched HIV-negative control group. Structures of the basal ganglia (caudaute nucleus, putamen, nucleus accumbens and ventral pallidum), the hippocampus, amygdala and thalamus were analyzed with a new algorithm based on a sensitive Bayesian method to estimate resting-state functional connectivity.

## Methods

### Participants

Participants who underwent MR examination were selected from the original cohort of the Art-NeCo study, a prospective Dutch cohort study on *Ne*uro*Co*gnition in HIV-infected patients on long term effective c*ART* (Janssen et al. 2015). Consecutive patients were recruited through their treating physicians at the participating centers, that is, Radboud University Medical Center in Nijmegen and the Rijnstate Hospital Arnhem. For inclusion, HIV-infected patients had to be on cART for at least one year with an HIVRNA in plasma <20 copies/ml for at least one year. Exclusion criteria for the patient group included active opportunistic infections, pregnancy, malignancy or neurosyphilis. Healthy HIV-negative control participants were matched on age, sex and educational level. Both patients and controls had to be between 18 and 70 years of age and fluent in Dutch language. Exclusion criteria for both patients and controls were current recreational drug or alcohol abuse, and a history of psychiatric or neurological disorder (unrelated to HIV infection in the patient group). HIV-1 status of patients was determined by enzyme-linked immunosorbent assays (ELISA) and a Western Blot confirmatory test, and with a rapid HIV test in healthy controls. Medical ethical approval for this study was obtained from the Medical Review Ethics Committee region Arnhem-Nijmegen (CMO #2011/267) and written informed consent was obtained from all participants.

Rs-fMRI data were acquired from 87 HIV-infected patients and 42 matched controls between January 2012 and January 2014. Fifteen patients and 3 controls were removed from analyses; two patients due to medical reasons unrelated to their HIV status, one due to enlarged ventricles secondary to atrophy and periventricular white-matter lesions, resulting in problems during registration and with FIRST, and 12 because of poor image quality resulting in low contrast in subcortical regions which was unrelated to HIV-infection. One control was removed from analyses due to scanner artifacts and two due to poor image quality resulting in low contrast in subcortical regions. All scans were visually screened by two radiologists (SCS and BJ) for relevant clinical abnormalities. Also, all processed scans were visually checked for errors in the preprocessing stages. The total sample therefore consisted of 72 patients and 39 controls. Note that based on neurocognitive testing, 27 of these patients fulfilled the criteria for asymptomatic neurocognitive impairment and 4 for mild neurocognitive disorder. None of the patients fulfilled the criteria for HIV-associated dementia.

### Imaging methods

MR examinations were performed on a 3 T Magnetom Trio scanner (Siemens, Erlangen, Germany). Whole-head structural MR images were acquired with a 3D T1-weighted MP-RAGE sequence (inversion time (TI) = 1000 ms, repetition time (TR) = 2300 ms, echo time (TE) = 4.71 ms, 12° flip-angle, field of view (FOV) = 256 mm, 192 sagittal slices, slice thickness = 1 mm, voxel-size = 1 × 1 × 1 mm^3^). Resting state data were acquired using a gradient-echo planar imaging (EPI) sequence (TR = 2380 ms, TE = 30 ms, flip angle (FA) = 90°, 41 slices, 64 × 64 matrix, voxel size =3.5 × 3.5 × 3 mm^3^, 110 volumes, Field of view (FoV) = 224 mm). All participants were scanned on the same scanner and were instructed to close their eyes, think of nothing in particular, and relax while avoiding falling asleep.

### Data preprocessing

The MRI datasets were preprocessed using FSL 5.0 using default settings unless noted otherwise. Structural scans were processed with FAST (Zhang et al. 2001) to obtain an estimate of, and correct for, the bias field. Brain extraction was performed on the corrected images using BET (Smith 2002), the output of which was processed again with FAST, this time to perform segmentation into white matter, gray matter and cerebrospinal fluid (CSF). Subcortical regions were parcellated using FIRST (Patenuade et al. 2011). Functional scans were motion corrected using MCFLIRT (Jenkinson et al. 2002) and linearly registered to the T1 image using FLIRT. Mean white matter and CSF signals, combined with the estimated motion and temporal derivatives thereof, were regressed out using FEAT. Finally, data were high-pass filtered with a cut-off of 100 Hz.

### Data analysis

Functional connectivity was determined between fourteen regions of interest (ROI): the left and right nucleus accumbens, amygdala, striatum, hippocampus, pallidum and thalamus, to investigate subcortical resting-state functional connectivity. A Bayesian method was used to estimate connectivity (Hinne et al. 2015), quantified in terms of partial correlations. This approach determines the probability distribution over all different network structures that are supported by the data, rather than estimating only the most probable network. The method is able to find unbiased functional connectivity and has been demonstrated to outperform state-of-the-art alternatives such as the graphical lasso (Friedman et al. 2008) in recovery of functional connectivity (Hinne et al. 2015). The method assumes that partial correlations, representing the strength of direct functional interactions, presuppose a skeleton of conditional independencies. That is, functional connectivity is represented by a binary network indicating *which* regions are connected, in addition to a weighted network of partial correlations that indicate the *strength* of connections. As partial correlations only need to be estimated for conditionally dependent pairs of ROI, the method reduces the effective number of parameters to be estimated without underestimating connection strengths. This is particularly effective when the number of samples is limited compared to the number of connections that must be estimated (Hinne et al. 2015).

The posterior distribution of functional connectivity (both the binary skeleton and the partial correlations) was estimated for each subject, using a Markov chain Monte Carlo (MCMC) technique. Subsequently, the posterior expectation of network density (of the binary graph) and the partial correlation strengths were computed to summarize the results per subject.

## Results

The groups were well balanced for age, sex and education level (see Table 1).

**Table 1:** Demographic variables for patients and controls

Characteristic	Patients (*N* = 72 )	Controls (*N* = 39)
Age (years) [mean (range, SD)]	48.8 (26–70, 9.3)	52.6 (28–68, 10.7)
Sex	61 (84.7 %) men	31 (79.5 %) men
	MSM: 55 (90.2 %)	MSM: 8 (25.8 %)
	11 (15.3 %) women	8 (20.5 %) women
Nadir CD4 cell count (cells/μL) [mean (IQR)]	213 (90–310)	n.a.
Duration HIV-infection (years) [mean (IQR, SD)]	9.4 (4.4–14.6, 6.4)	n.a.
Duration cART treatment (years) [mean (IQR, SD)]	7.8 (3.1–12.8, 5.6)	n.a.
Regular alcohol use^a^	17 (23.6 %)	20 (51.3 %)
Regular drug use^b^	7 (9.6 %)	3 (7.7 %)
Education level [median (range)]^c^	6 (2–7)	6 (3–7)
Estimated IQ [mean (SD)]	98^*^(14.5)	104 (14.0)

*n.a.* not applicable, *MSM* Men who have sex with men

^a^Regular alcohol use is use of alcohol for three or more times a week or binge drinking on two subsequent days

^b^Regular drug use is use of a drug for four times or more times a month

^c^Education level was recorded using seven categories based on the Dutch educational system, which can be related to the Anglo-Saxon system using years of education: 1:1–5 years, 2:6 years, 3:7–8 years, 4:7–9 years, 5:7–10 years, 6:7–17 years and 7: >18 years

^*^ *p* < 0.05

### Partial correlations

**Fig. 1:**
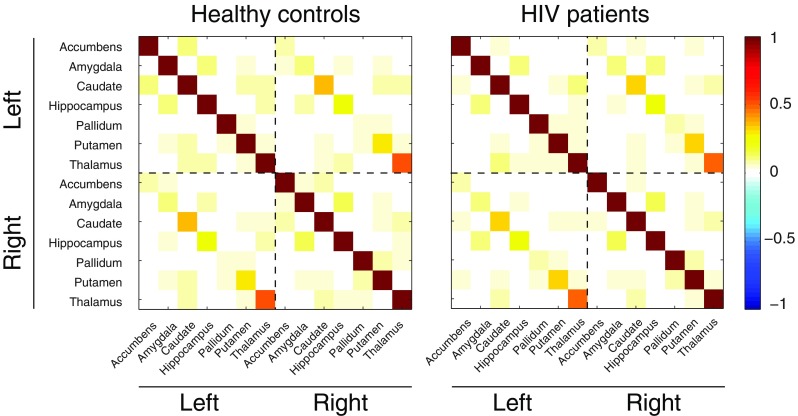
Functional connectivity estimates for the healthy control group and HIV-infected patients

**Fig. 2:**
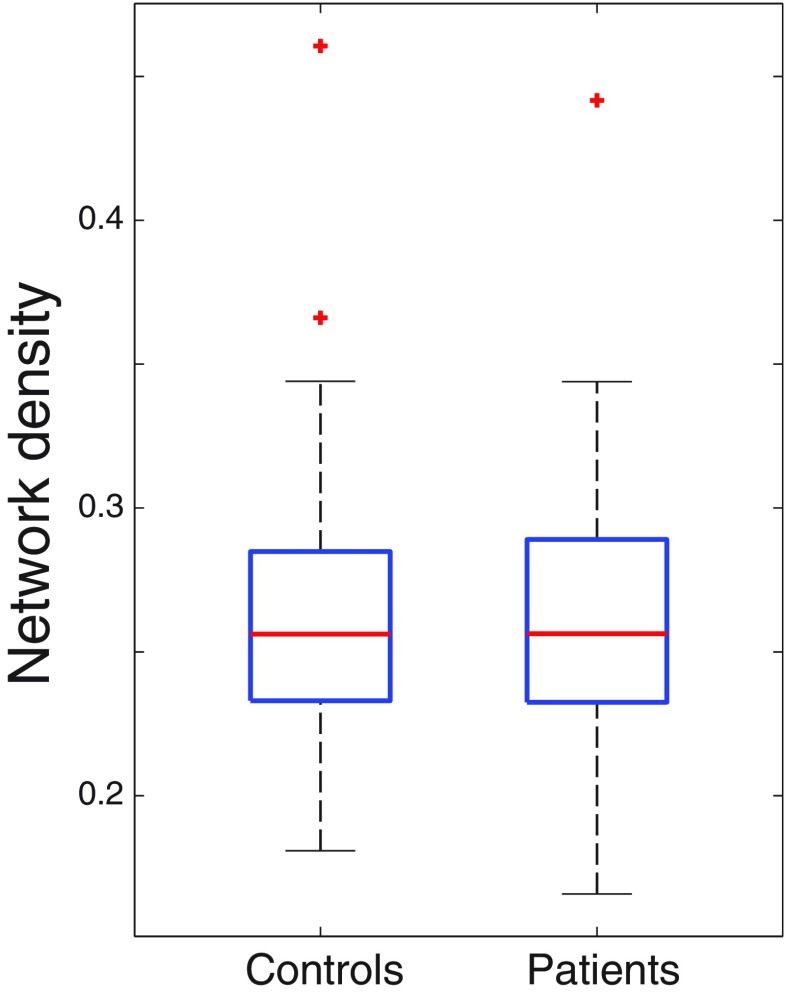
Network density for the healthy control group and HIV-infected patients

The connectivity estimates, quantified in terms of partial correlations, are shown as adjacency matrices in Fig. 1, for both the healthy control group and the HIV-infected patients. In these plots, each element of the matrix indicates the expected partial correlation between the row-ROI and the column-ROI, averaged over all participants per group. These partial correlations were in the range [−0.04, 0.55] for the control group and in the range [−0.05, 0.50] for the patient group. As the matrices indicate, both groups show the strongest partial correlations between the homotopic regions in the different hemispheres, as indicated by the diagonals in the upper right and lower left quadrants. The strongest partial correlations in both groups are found between the left and right caudate nucleus (0.41, SD = 0.01 for the control group and 0.36, SD = 0.01 for the patient group) and the left and right thalamus (0.55, SD = 0.01 for the control group and 0.50, SD = 0.01 for the patient group). However, no substantial differences appear to be present between the two groups upon visual inspection of the matrices. This was confirmed by computing the posterior expected network densities for all participants per group, between which, again, no significant difference was found (control network density = 0.26, SD = 0.05, patient network density = 0.26, SD = 0.04*, p = 0.58*, 2-tailed t-test) (Fig. 2).

## Discussion

This is the first study that has used a new algorithm based on a sensitive Bayesian method to estimate resting-state functional connectivity between fourteen subcortical regions of interest in HIV-infected patients compared to a well-matched healthy control group. Our data show the strongest partial correlations between the homotopic regions in the two hemispheres and particularly between the left and right caudate and the left and right thalamus. However, no differences in the posterior expected network densities were found between the HIV-infected patients and the healthy control group.

The results of the current study are not in agreement with earlier rs-fMRI findings showing reduced connectivity between various brain networks in HIV-infected patients compared to healthy controls, as we did not find any differences in subcortical connectivity between patients and controls (Ipser et al. 2015; Thomas et al. 2013; Wang et al. 2011; Ortega et al. 2015). One important difference between the current study and the earlier studies is that in our study only patients on cART and with long term undetectable HIVRNA (at least one year) were included, while in the other studies more heterogeneous HIV groups were used. Furthermore, previous studies focused on subcortical-cortical connectivity, while in the current study subcortical connectivity was investigated. Volumetric subcortical atrophy has been reported in earlier studies (Ances et al. 2012; Cohen et al. 2010), also in HIV-infected patients with well-controlled immune status and viral replication (Becker et al. 2011). Moreover, Thames et al. (2016) showed increased activation in the putamen and caudate nucleus during a lexical retrieval task.

Our findings are in line with the finding of Ortega et al. (2015), who showed that patients on cART had higher functional connections within the ventral attention network than patients who were not on cART. Also, their patients on cART had similar functional connections in the default mode network as the HIV negative control group. Successful cART has been shown to reduce glial activation as well as inflammation caused by HIV infection (Young et al. 2014; Sailasuta et al. 2012). Moreover, cART decreases HIV viral load in the central nervous system (CNS) and reduces the synapto-dendritic damage that occurs by HIV (Crews et al. 2009; Masliah et al. 1997). Therefore, as cART reduces inflammation in the CNS it may improve synaptic communication, which may in turn lead to normalized connectivity in various resting-state networks.

One could argue that a relatively small number of samples (110) were acquired for the resting-state fMRI scan in relation to the number of estimated connections (91) in the current study, limiting the signal-to-noise (SNR) ratio. Clearly, more volumes result in a better SNR-ratio, which increases the likelihood of detecting small differences between groups. Therefore, it remains to be investigated whether our conclusions are maintained in the face of larger sample sizes. Also, our study sample consisted of patients and controls in a broad age range (i.e., 18–70). Although the age range was comparable for patients and controls, future studies should specifically examine the role of age and functional connectivity in HIV. That is, while aging in HIV may be an independent factor (Thomas et al. 2013), others reported interactions between aging and HIV-related changes in functional connectivity (Jahanshad et al. 2012). With our current sample, stratification into multiple age groups would have resulted in insufficient statistical power due to small subgroups. Also, the heterogeneity with respect to duration of the HIV infection, cART treatment duration and CD4 count should be examined using larger samples, as these disease markers are associated with disruptions in resting state functional connectivity (Guha et al. 2016). The Bayesian approach of the present study, however, requires stratified groups to take these potentially confounding factors into account.

Previous results from the Art-NeCo study showed small but significant volume differences in the thalamus and total brain volume between HIV-infected patients and controls, with the patient group showing smaller volumes. In that study, no effect of HIV infection was found on overall cognitive performance. In the present study no differences in resting-state subcortical connectivity were found between the HIV-infected patients and the healthy control group, which adds to recent findings (Janssen et al. 2015; Su et al. 2015; Heaton et al. 2010) showing only small cognitive decrements and neural changes in successfully treated HIV-infected patients on long-term cART who are otherwise healthy. However, this result should be interpreted with some caution, as we used a novel algorithm to analyze our data. Validation of our findings is needed, using larger samples in order to control for various confounding factors like age and disease variables.
